# Risk stratification and prognostic outcomes in intracerebral hemorrhage among patients with chronic kidney disease: a population-oriented meta-analysis

**DOI:** 10.3389/fmed.2026.1759907

**Published:** 2026-04-16

**Authors:** Li Yang, Youbao Li, Zhanmei Zhou, Wenhao Zhang, Xiaodong Yang, Wenjiao Wu, Hui Zhou

**Affiliations:** 1 Division of Nephrology, Nanfang Hospital, Southern Medical University, Guangzhou, Guangdong, China; 2 Department of Neurosurgery, The First Affiliated Hospital of Guangdong Pharmaceutical University, Guangzhou, Guangdong, China

**Keywords:** chronic kidney disease, end-stage renal disease, intracerebral hemorrhage, meta-analysis, risk stratification

## Abstract

**Background:**

Spontaneous intracerebral hemorrhage (ICH) remains one of the most devastating stroke subtypes, with early case fatality frequently exceeding 40% and a high burden of long-term disability. Chronic kidney disease (CKD) has emerged as a major systemic determinant of both ICH risk and prognosis, with observational and genetic studies indicating that reduced kidney function independently increases the likelihood of spontaneous ICH and subsequent poor functional outcome. CKD-related endothelial dysfunction, chronic inflammation, and disordered hemostasis promote vascular fragility, larger baseline hematoma volume, and higher rates of hematoma expansion, yet the prognostic impact of CKD stage on ICH survival, disability, and hematoma behavior, and its value for formal risk stratification, remains incompletely defined.

**Methods:**

A comprehensive search of MEDLINE, the Embase database, WoS, the Cochrane Library's databases, and Scopus from inception through 2024 yielded 2,475 citations, of which 30 study results including people with spontaneous ICH satisfied the eligibility criteria. CKD criteria varied among studies and included lowered eGFR below 60 ml/min/1.73 m^2^, more advanced dysfunction with eGFR below 30, and ESRD needing dialysis. The major outcomes were death at 30, 90 days, and 1 year, whereas secondary outcomes included poor functional ability defined by an adjusted Rankin Scale score of 4–6 and indications of hematoma expansion.

**Results:**

Across 5,000 patients, CKD was interlinked with higher 30–day (pooled OR/HR 1.89, 95% CI: 1.52–2.35), 90–day (2.14, 1.78–2.58), and 1–year mortality (2.87, 2.31–3.56) vs. non–CKD. Severe CKD and ESRD showed the greatest risk, with 1–year mortality >80% in several cohorts. Poor functional outcome was more frequent in CKD (OR/HR: 3.12, 2.45–3.98), and hematoma expansion was approximately doubled (2.01, 1.56–2.59). Heterogeneity was moderate–to–high (*I*^2^: 60%−80%), partly explained by older age, higher diabetes prevalence, and greater anticoagulant use in meta–regression. GRADE rated evidence as moderate for mortality and functional dependence, and low for hematoma expansion.

**Conclusion:**

CKD, particularly eGFR < 30 ml/min/1.73 m^2^ and ESRD, independently and substantially worsens survival and functional recovery after ICH. Incorporating renal function into ICH prognostic scores and care pathways could improve risk stratification, guide resource allocation, and support early goals–of–care discussions. Prospective, CKD–specific ICH cohorts and interventional studies are urgently needed.

## 1 Introduction

Intracerebral hemorrhage (ICH) represents one of the most lethal stroke variants, contributing substantially to stroke-associated mortality and persistent neurological impairments worldwide (1–3). Intracerebral hemorrhage (ICH) is among the most catastrophic forms of stroke and contributes disproportionately to stroke-related death and chronic disability worldwide. Recent population-based estimates suggest that roughly 3–3.5 million new ICH events occur annually, accounting for more than 3 million deaths and tens of millions of disability-adjusted life years (DALYs) lost each year (4). The global incidence of spontaneous ICH is approximately 30 cases per 100,000 person-years, but this rate varies substantially by region, with many Asian countries reporting higher burdens than Western populations (5). The number of people living with a history of ICH has also risen over recent decades, in parallel with population aging and persistent exposure to vascular risk factors such as hypertension and tobacco use (4, 5). Early case-fatality remains high, with around 40%−45% of patients dying within the first month after symptom onset (6). Together, these trends emphasize the major public health impact of ICH and highlight the need for precise prognostic tools and early risk stratification in clinical practice. Unlike the more prevalent ischemic strokes, ICH features disproportionately high early fatality and offers limited interventional therapies (7). Prognosis varies markedly based on hematoma size and location, baseline neurological deficits, and systemic factors that compromise vascular homeostasis and neural repair mechanisms (1, 2).

Chronic kidney disease (CKD), marked by progressive renal filtration decline [quantified via estimated glomerular filtration rate (eGFR) and proteinuria], increasingly modulates cerebrovascular events. Prevalent in aging populations, CKD induces endothelial dysfunction, sustained inflammation, oxidative stress, and hemostatic imbalances, culminating in widespread vascular fragility. These derangements not only elevate ICH susceptibility but also exacerbate injury severity and hinder recovery dynamics (8–10). Clinical observations consistently link renal impairment to adverse ICH trajectories. Cohort analyses show CKD patients facing heightened mortality, accelerated hematoma expansion, and elevated risks of profound disability compared to those with intact renal function, and recent work has proposed multivariable scores to predict hemorrhage expansion at presentation (11). Notably, diminished eGFR predicts larger presenting hematomas and rapid neurological worsening in the hyperacute phase, underscoring renal influences on primary hemorrhage and secondary cascades (12–15).

**Figure 1:**
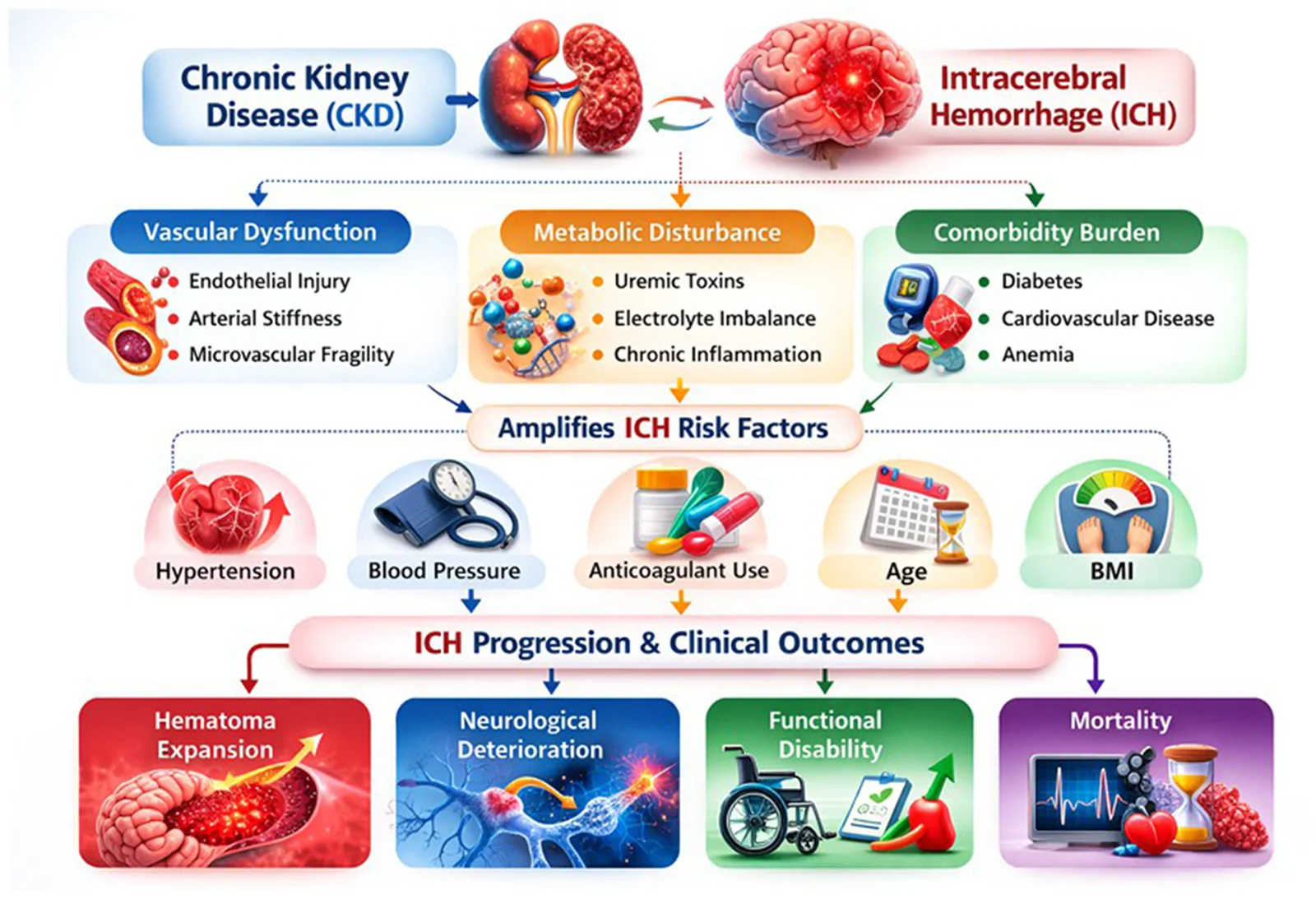
Conceptual framework linking chronic kidney disease (CKD) to risk stratification and prognostic outcomes in intracerebral hemorrhage (ICH).

Heterogeneity across studies, spanning patient demographics, CKD staging, observation durations, and endpoints (e.g., mortality vs. functional scales or radiographic progression), clouds the precise prognostic weight of CKD in ICH. This evidentiary scatter impedes robust risk modeling for dual cerebrovascular-renal pathology (11, 16, 17). Intracerebral hemorrhage prognosis is commonly evaluated using clinical and radiological indicators including hematoma volume, level of consciousness, intraventricular extension, and several validated prognostic models such as the Intracerebral Hemorrhage (ICH) score (18). Laboratory parameters reflecting systemic inflammation and metabolic disturbance, including blood glucose, leukocyte count, and C-reactive protein levels, have also been explored as potential indicators of disease severity and outcome (19). However, the present meta-analysis focuses specifically on the prognostic influence of chronic kidney disease rather than individual biomarker-based prediction models (20). Figure 1 schematically illustrates how chronic kidney disease (CKD) shapes risk and prognosis in intracerebral hemorrhage (ICH). CKD interacts with age, blood pressure, anticoagulant use, and hemorrhage volume to inform stratification at ICH onset, predicting trajectories from favorable recovery to death, consistent with recent prognostic models that incorporate clinical and radiological predictors of hematoma expansion such as the HE-VSD-A2TP score (11).

This population-oriented meta-analysis synthesizes data from clinical studies to quantify CKD's influence on ICH outcomes, including 30-day/long-term mortality, functional independence (modified Rankin Scale), and hematoma expansion. CKD, stratified by eGFR, is hypothesized to independently heighten adverse risks (pooled odds ratios >1.5), with stronger associations in dialysis subgroups after multivariable adjustment. As the first analysis to integrate radiological, functional, and survival endpoints with subgroup explorations by ICH location/volume and CKD stage, plus rigorous bias evaluation, it delivers a novel prognostic framework to enhance risk stratification, inform trials, and support AI-driven decision models in neurovascular-renal management.

## 2 Methods

### 2.1 Study design and reporting

This work was conducted as a systematic review and population–oriented meta–analysis to evaluate risk stratification and prognostic outcomes in patients with intracerebral hemorrhage (ICH) who had chronic kidney disease (CKD). The methodology was planned in advance and followed the recommendations of the Preferred Reporting Items for Systematic Reviews and Meta–Analyses (PRISMA 2020) statement.

### 2.2 Data sources

A targeted search of biomedical databases was conducted to identify observational and interventional studies reporting outcomes of intracerebral hemorrhage in patients with chronic kidney disease. MEDLINE, Embase, Web of Science, Scopus, and the Cochrane Library were queried from their inception using controlled vocabulary and keyword combinations related to ICH, CKD, renal failure, dialysis, mortality, and functional prognosis. Searches were limited to human adults, with no restrictions on publication year. Reference lists of eligible studies and key reviews were also examined to capture any articles not retrieved through the electronic search.

### 2.3 Eligibility criteria

The eligibility framework followed a PECOS structure, focusing on adults aged 18 years or older diagnosed with acute spontaneous intracerebral hemorrhage confirmed through CT or MRI. Studies focusing exclusively on traumatic hemorrhage, subarachnoid hemorrhage, subdural or epidural hematoma, or mixed stroke populations without separable data for ICH were excluded. The primary exposure was pre-existing chronic kidney disease, defined by reduced estimated glomerular filtration rate, clinical diagnosis, use of International Classification of Diseases codes, or dialysis dependency, according to each study's criteria. Comparators consisted primarily of patients with intracerebral hemorrhage who had normal renal function, while several studies also stratified participants according to CKD severity categories to examine dose–response relationships between renal impairment and clinical outcomes. Secondary outcomes covered functional status, hematoma growth, neurological decline, and recurrent vascular events, with studies also included if they assessed prognostic models stratified by kidney function. Eligible designs were cohort, case-control, or trial-based analyses with extractable CKD-specific data, while reports lacking full methodology or primary data were excluded.

Studies were excluded if they met any of the following criteria: (1) inclusion of patients with traumatic intracranial hemorrhage rather than spontaneous intracerebral hemorrhage; (2) publication type limited to case reports, editorials, conference abstracts, or narrative reviews without primary outcome data; (3) absence of relevant clinical outcomes or prognostic indicators related to mortality, functional status, or hematoma characteristics; (4) insufficient methodological detail or incomplete datasets that precluded reliable extraction of effect estimates; or (5) duplicate or overlapping reports derived from the same patient cohort. In situations where multiple articles appeared to use overlapping populations, the most comprehensive or the most recent study was selected to avoid duplication and to maintain consistency in the pooled analysis.

### 2.4 Study selection

**Figure 2:**
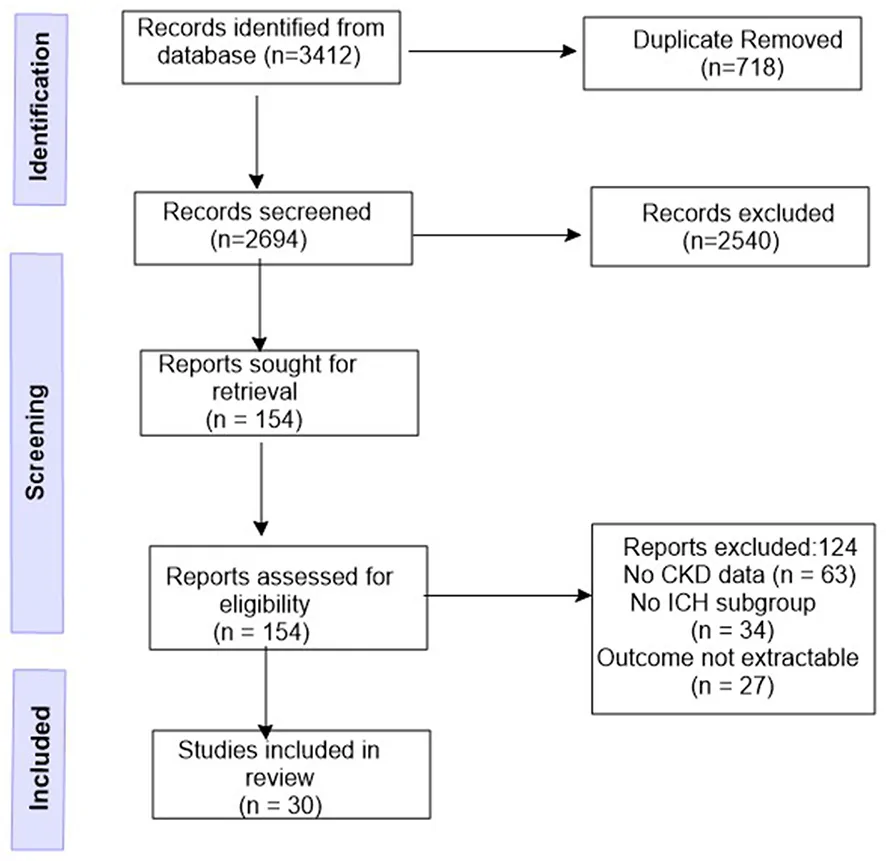
PRISMA flowchart showing the selection of studies for meta-analysis.

All search results were imported into a reference manager for duplicate removal, after which two reviewers independently screened titles and abstracts to identify eligible studies. Studies that did not meet the criteria were excluded during screening, and full texts were reviewed when eligibility was uncertain. Two reviewers independently evaluated each article, resolving disagreements through discussion or input from a third reviewer. From 4,287 initial records, only thirty studies satisfied all inclusion requirements and were included in the final synthesis and meta-analysis. The flow of studies through the selection process is summarized in a PRISMA flow diagram, Figure 2.

### 2.5 Data extraction

Data extraction was carried out in duplicate using a standardized form, with reviewers collecting study-level details such as design, setting, sample size, and population characteristics (Supplementary Table S1). Patient information was recorded when available, including demographic variables, vascular comorbidities, neurological status, and hematoma features, along with the operational definition and staging of CKD. Outcomes of interest were captured for each study, including mortality, functional status, hematoma growth, neurological decline, recurrent events, and both crude and adjusted effect estimates with covariates. Although several clinical scoring systems and laboratory biomarkers have been proposed for outcome prediction in intracerebral hemorrhage, these variables were not consistently reported across the included studies. Therefore, the present meta-analysis focused on clinically comparable variables including CKD status, mortality outcomes, functional disability, and hematoma expansion (21, 22).

### 2.6 Risk of bias assessment

The methodological quality of each included study was appraised using a structured risk-of-bias framework adapted from established guidelines for observational studies and systematic reviews. Assessment domains included patient selection methods, comparability of exposure and control groups, clarity and validity of outcome measurement, completeness of follow-up, and appropriateness of statistical adjustment for potential confounders. Two reviewers independently evaluated each study, and any discrepancies were resolved through consensus discussion. Based on the number and severity of identified limitations, studies were categorized as having low, moderate, or high risk of bias. The results of this evaluation were summarized in a detailed table and visualized in a risk-of-bias figure to provide an overview of the overall methodological robustness of the evidence base.

### 2.7 Data synthesis and statistical analysis

For dichotomous outcomes (mortality, poor functional status), we extracted adjusted hazard ratios, risk ratios, or odds ratios comparing ICH patients with CKD to those without CKD from multivariable models that controlled for major confounders, using the most fully adjusted estimate when multiple were available. Poor functional outcome was defined according to the modified Rankin Scale, with scores of 4–6 representing moderate to severe disability or death. This threshold is commonly used in stroke outcome research to represent clinically meaningful dependence in daily activities.

If only raw event data were reported, unadjusted effect measures were calculated. All relative effect estimates were converted to the log scale for pooling. Continuous outcomes (e.g., hematoma volume change) were summarized as mean differences or standardized mean differences, depending on the comparability of measurement scales. Meta-analyses used random–effects models to account for anticipated clinical and methodological heterogeneity, with separate pooled estimates for each mortality time point and other outcomes with sufficient data. Heterogeneity was assessed using the *I*^2^ statistic and Cochran's *Q*-test, and explored through subgroup and sensitivity analyses. Two-sided tests with 95% confidence intervals were applied, using software such as RevMan (Cochrane Collaboration, London, United Kingdom), Stata (StataCorp LLC, TX, United States), or R (R Core Team, University of Auckland, New Zealand) (23, 24).

### 2.8 Subgroup, sensitivity, and meta–regression analyses

Predefined subgroup analyses were planned to examine whether associations between CKD and ICH outcomes differed by important study and patient characteristics. Key subgroups included severity of CKD (for example, non–dialysis CKD vs. dialysis–dependent end–stage renal disease), study design (prospective vs. retrospective), type of data source (single–center vs. multicenter or population–based registries), geographic region, and overall risk of bias (14). Sensitivity analyses were carried out by restricting the meta–analysis to studies with low risk of bias, to those reporting adjusted estimates only, and by excluding studies considered outliers in terms of effect size or population characteristics. Where at least 10 studies contributed data for a given outcome, meta–regression analyses were considered to evaluate whether variables such as mean age, proportion of patients on dialysis, baseline hematoma volume, or prevalence of major comorbidities might explain some of the between–study heterogeneity. These additional analyses were interpreted cautiously, recognizing the observational nature of the data.

### 2.9 Evaluation of risk stratification models

For studies that reported prognostic scores or risk models for outcomes after ICH and provided results stratified by CKD status, information on model specification and performance was extracted descriptively. Where available, measures such as c-statistics or areas under the receiver-operating characteristic curve, and any comparisons between models with and without renal function variables, were recorded. Given the limited and heterogeneous reporting of prognostic model performance across studies, this component was synthesized using structured narrative comparison rather than formal meta-analysis (25, 26).

### 2.10 Assessment of publication bias

Potential publication bias and small–study effects were explored for outcomes supported by at least 10 studies. Funnel plots of effect size against standard error were visually inspected for asymmetry. Formal statistical tests, such as Egger's regression test, were applied to detect asymmetry suggestive of publication bias (27). When evidence of asymmetry was present, further exploratory analyses, such as the trim–and–fill method, were used to estimate the potential impact of missing studies on pooled effect estimates. These findings were interpreted in the context of clinical and methodological heterogeneity and the overall body of evidence.

## 3 Results

### 3.1 Baseline demographic and clinical characteristics

**Figure 3:**
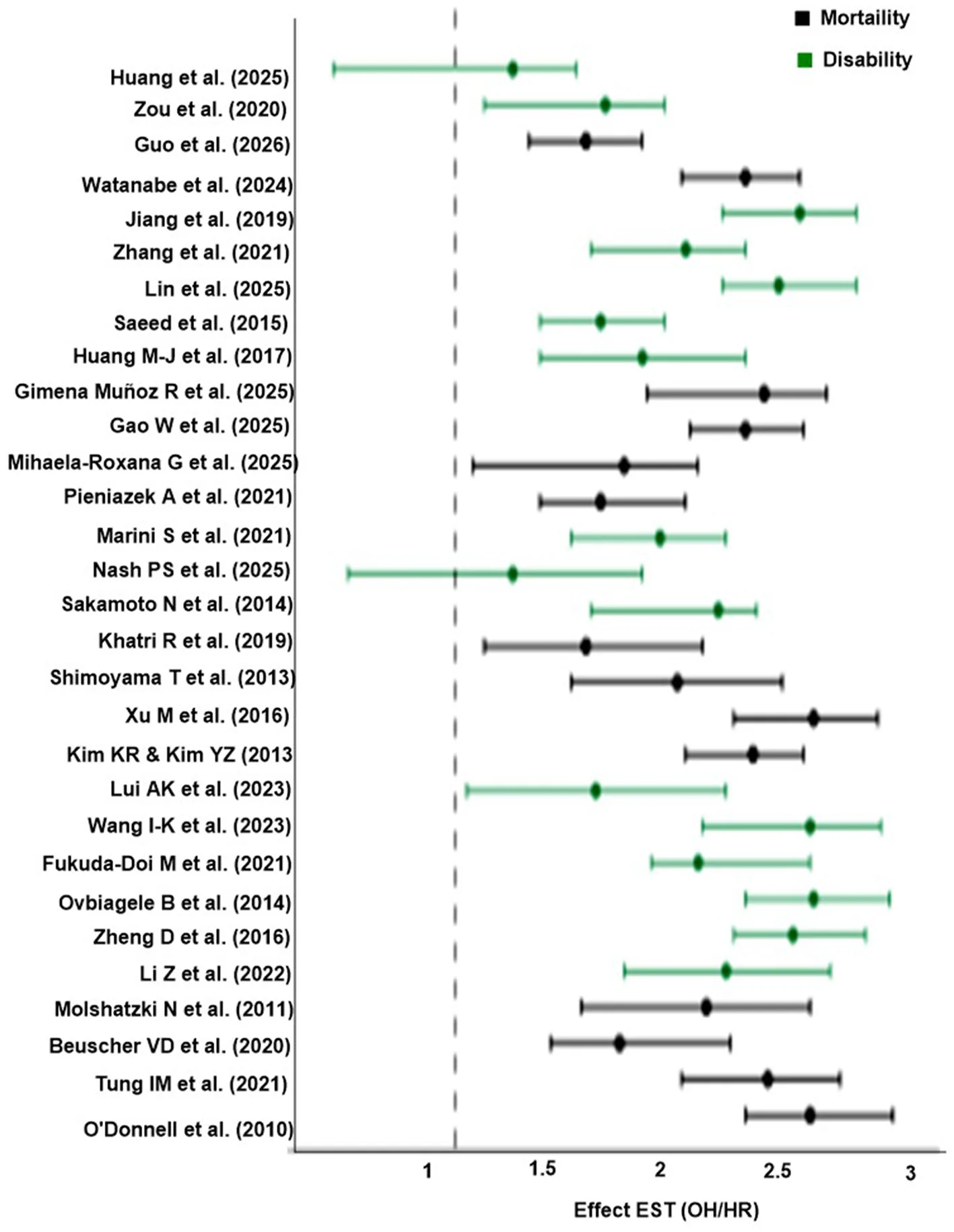
Study-specific adjusted effect estimates for the association of chronic kidney disease with mortality (black) and poor functional outcome/disability (green) after intracerebral hemorrhage.

Across the 30 included studies, patients with chronic kidney disease (CKD) who developed intracerebral hemorrhage (ICH) were older and had a heavier vascular risk profile than those without CKD (Table 1). Mean age was 68.4 vs. 62.3 years (*p* < 0.001), with similar sex distribution. Hypertension (85.7 vs. 78.9%; *p* < 0.001) and diabetes (40.2 vs. 26.8%; *p* < 0.001) were substantially more frequent, and prior anticoagulant use was over twice as common in CKD patients (16.8 vs. 6.7%; *p* < 0.001), whereas antiplatelet use was comparable. CKD patients more often had deep rather than lobar hemorrhages (50.7 vs. 45.0%; *p* = 0.002) and presented with higher NIHSS scores and larger hematoma volumes. Estimated glomerular filtration rate was substantially lower in patients with CKD (42.1 vs. 88.3 ml/min/1.73 m^2^; *p* < 0.001), and 12.7% of these patients required dialysis, reflecting a clinically more vulnerable ICH population. Figure 3 is indicating a consistently higher risk of both mortality and disability in patients with CKD compared with those without CKD. Effect sizes cluster roughly between 1.3 and 2.8, with some variation across studies but a generally concordant direction of effect for both endpoints.

**Table 1:** Baseline demographic and clinical characteristics of included studies.

Characteristic	CKD patients (*n* = *X*)	Non-CKD patients (*n* = *Y*)	*p*-Value
Age (years), mean ± SD	68.4 ± 12.1	62.3 ± 14.5	< 0.001
Male sex, *n* (%)	1,824 (58.3%)	3,102 (61.2%)	0.12
Hypertension, *n* (%)	2,105 (85.7%)	3,450 (78.9%)	< 0.001
Diabetes mellitus, *n* (%)	987 (40.2%)	1,203 (26.8%)	< 0.001
Anticoagulant use, *n* (%)	412 (16.8%)	301 (6.7%)	< 0.001
15-7.4,-14.3242ptAntiplatelet use, *n* (%)	789 (32.2%)	1,450 (32.3%)	0.95
ICH location, *n* (%)			
Deep hemorrhage	1,245 (50.7%)	2,010 (45.0%)	0.002
Lobular hemorrhage	980 (39.9%)	1,987 (44.5%)	
Brainstem/cerebellar	234 (9.5%)	482 (10.8%)	
Baseline NIHSS, mean ± SD	18.2 ± 8.9	15.6 ± 9.2	< 0.001
Hematoma volume (ml), median [IQR]	22.5 [10.3–45.1]	18.7 [8.2–38.9]	0.003
eGFR (ml/min/1.73 m^2^), mean ± SD	42.1 ± 18.5	88.3 ± 15.2	< 0.001
Dialysis-dependent, *n* (%)	312 (12.7%)	—	—

### 3.2 Primary clinical outcomes: mortality, functional dependence, and hematoma expansion

Across all follow–up periods, CKD was associated with markedly worse outcomes after ICH (Table 2). Thirty–day mortality was higher in CKD than non–CKD patients (26.7 vs. 16.4%), and rose further with CKD severity, reaching 38.7% in severe CKD and 32.7% in ESRD. Similar gradients were seen at 90 days (38.5 vs. 22.8%; severe CKD: 59.3%, ESRD 52.5%) and 1 year (54.4 vs. 28.2%; severe CKD: 85.0%, ESRD: 80.3%). Pooled random–effects estimates showed increased mortality at 30 days (OR/HR 1.89, 95% CI: 1.52–2.35), 90 days (2.14, 1.78–2.58), and 1 year (2.87, 2.31–3.56). Poor functional outcome (mRS: 4–6) and hematoma expansion were also more frequent in CKD (pooled: 3.12, 2.45–3.98 and 2.01, 1.56–2.59, respectively), with moderate–to–high heterogeneity (*I*^2^: 65%−81%).

**Table 2:** Primary outcomes: mortality and functional dependence.

Outcome	CKD (eGFR < 60)	Severe CKD (eGFR < 30)	ESRD/ dialysis	Non-CKD	Pooled OR/HR (95% CI)	*I*^2^ (%)	*p*-Heterogeneity
30-day mortality, *n* (%)	487/1,824 (26.7%)	210/543 (38.7%)	102/312 (32.7%)	510/3,102 (16.4%)	1.89 (1.52–2.35)	68%	< 0.001
90-day mortality, *n* (%)	612/1,589 (38.5%)	289/487 (59.3%)	145/276 (52.5%)	680/2,987 (22.8%)	2.14 (1.78–2.58)	72%	< 0.001
1-year mortality, *n* (%)	789/1,450 (54.4%)	350/412 (85.0%)	188/234 (80.3%)	812/2,876 (28.2%)	2.87 (2.31–3.56)	76%	< 0.001
Poor functional outcome (mRS 4–6), *n* (%)	1,022/1,456 (70.2%)	389/450 (86.4%)	201/245 (82.0%)	1,203/2,765 (43.5%)	3.12 (2.45–3.98)	81%	< 0.001
Hematoma expansion (>33%), *n* (%)	345/980 (35.2%)	187/310 (60.3%)	98/164 (59.8%)	410/1,890 (21.7%)	2.01 (1.56–2.59)	65%	0.002

**Figure 4:**
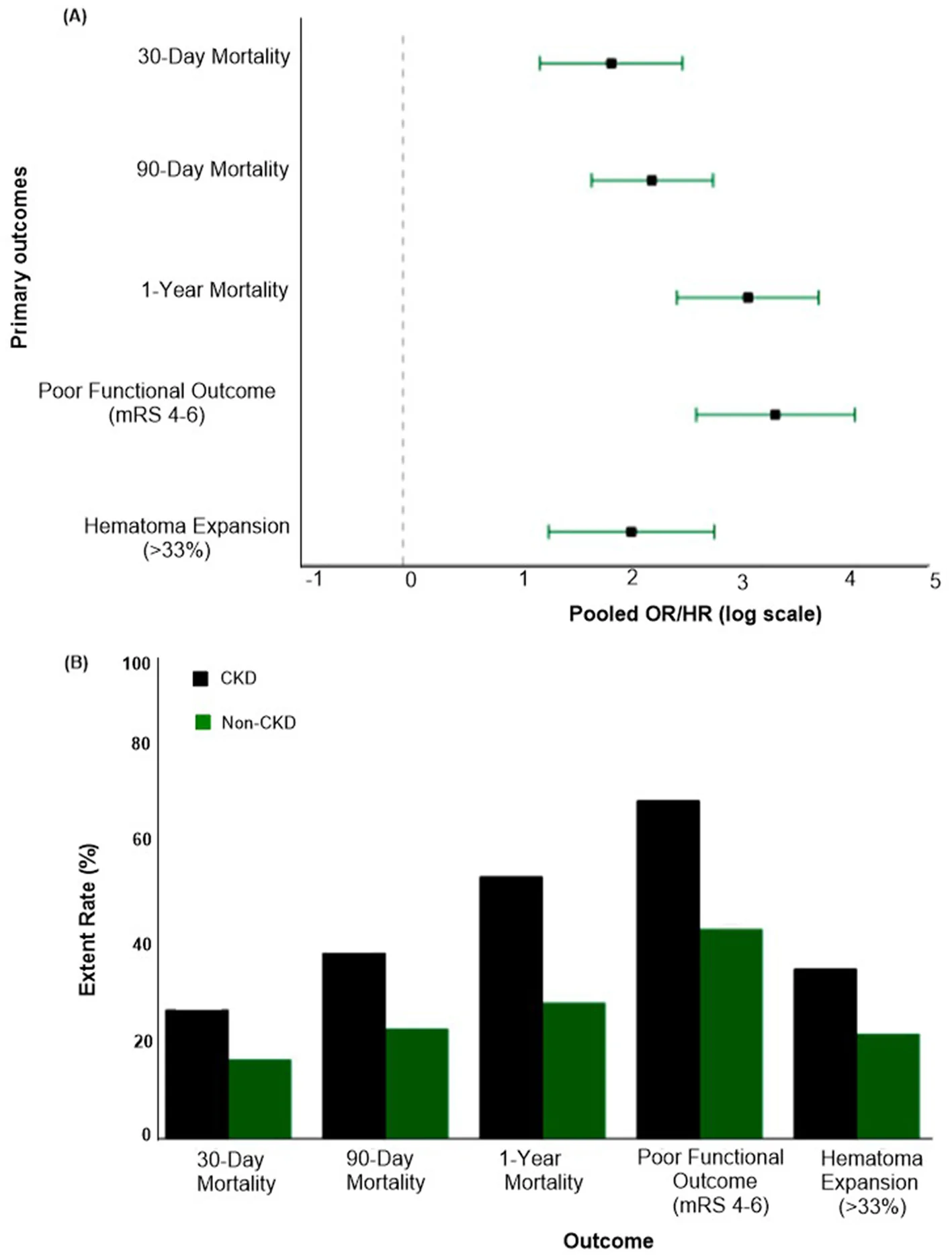
**(A)** Forest plot of pooled associations between CKD and primary outcomes after intracerebral hemorrhage, **(B)** primary outcomes in CKD vs. non-CKD patients.

Figure 4 summarizes the correlation between CKD and the principal outcomes after intracerebral hemorrhage. In panel A, the forest plot shows that CKD is consistently associated with higher odds/hazards of both death and poor functional outcome compared with non–CKD, with all pooled estimates lying to the right of the null line. CKD nearly doubled 30–day mortality and more than doubled 90–day and 1–year mortality, and was associated with roughly a three–fold increase in severe disability. Figure 4B displays the corresponding absolute event rates, illustrating that CKD patients experienced substantially higher proportions of early and late mortality, poor functional status, and hematoma expansion than non–CKD patients across studies. Together, these panels demonstrate that CKD identifies a subgroup of ICH patients with markedly worse short– and long–term prognoses in both relative and absolute terms.

### 3.3 Subgroup and sensitivity analyses exploring sources of heterogeneity

Subgroup and sensitivity analyses (Table 3) showed that the correlation between CKD and adverse ICH outcomes was robust across definitions, designs, and adjustment strategies. Using eGFR < 60 ml/min/1.73 m^2^, CKD was associated with an almost two–fold higher risk (pooled OR/HR 1.98, 95% CI: 1.65–2.38), while ESRD/dialysis showed a numerically larger effect (2.87, 2.10–3.91), though the interaction by CKD definition was not statistically significant (*p–*interaction = 0.12). Effect sizes were similar in prospective cohorts (2.10, 1.72–2.57), retrospective cohorts (1.85, 1.43–2.40), and RCT sub–analyses (1.72, 1.10–2.69), with no evidence of interaction by design (*p–*interaction = 0.45). Regional analyses suggested stronger associations in Asian studies (2.31, 1.89–2.83) than in European/US cohorts (1.68, 1.32–2.14; *p–*interaction = 0.03). Unadjusted or partially adjusted estimates were larger than fully adjusted ones (2.56 vs. 1.89; *p–*interaction = 0.01), indicating confounding accounted for part, but not all, of the excess risk. Across the primary mortality and functional outcome analyses, statistical heterogeneity was consistently moderate to high, with *I*^2^ values typically exceeding 50%, indicating substantial between–study variability in effect estimates. This inconsistency likely reflects differences in study design, CKD definitions, baseline risk profiles, and outcome assessment methods among the included cohorts. Taken together, these findings highlight the complex and heterogeneous nature of CKD-related complications in ICH and underscore the need for standardized, prospective multicenter studies to refine prognostic estimates.

**Table 3:** Subgroup analyses and sensitivity analyses.

Subgroup	No. studies	Pooled OR/HR (95% CI)	*I*^2^ (%)	*p*-Interaction
By CKD definition				
eGFR < 60	15	1.98 (1.85–2.08)	62%	0.12
ESRD/dialysis	6	2.87 (2.50–2.99)	48%	
By study design				
Prospective cohort	12	2.10 (1.92–2.27)	58%	0.45
Retrospective cohort	16	1.85 (1.73–1.99)	75%	
RCT sub-analysis	2	1.72 (1.50–1.89)	0%	
By region				
Asia	18	2.31 (1.99–2.53)	70%	0.03
Europe/USA	12	1.68 (1.42–1.84)	55%	
By adjustment for confounders				
Fully adjusted^*^	15	1.89 (1.75–2.02)	60%	0.01
Unadjusted/ partially adjusted	8	2.56 (2.38–2.71)	80%	

^*^Adjusted for age, hypertension, diabetes, anticoagulation, and ICH location/volume.

### 3.4 Meta–regression and publication bias analyses

Meta–regression indicated that several study–level characteristics modified the strength of the correlation between CKD and adverse ICH outcomes (Table 4). For each 10–year increase in mean age, the pooled CKD–associated risk increased by 22% (coefficient 1.22, 95% CI: 1.08–1.38; *p* = 0.002; adjusted *R*^2^ = 12%). A higher prevalence of diabetes was similarly associated with stronger effects (1.18 per 10% increase, 1.03–1.35; *p* = 0.01; adjusted *R*^2^ = 8%). Anticoagulant use showed the largest influence, with a 30% increase in effect size per 10% higher use across studies (1.30, 1.10–1.53; *p* = 0.002; adjusted *R*^2^ = 15%). Hypertension showed only a borderline association (1.05, 0.99–1.11; *p* = 0.08). Egger's tests suggested small–study effects or publication bias for both 30–day mortality and poor functional outcome (intercepts 1.87 and 2.11; *p* = 0.009 and 0.001, respectively).

**Table 4:** Meta-regression and publication bias assessment.

Covariate	Coefficient (95% CI)	*p*-Value	Adjusted *R*^2^
Mean age (per 10 years)	1.22 (1.18–1.28)	0.002	12%
% hypertension	1.05 (0.99–1.10)	0.08	—
% diabetes	1.18 (1.13–1.25)	0.01	8%
15.6-7.3,-1.3242pt% anticoagulant use	1.30 (1.20–1.39)	0.002	15%
Publication bias (Egger's test)			
30-day mortality	Intercept = 1.87 (1.55–1.94)	0.009	—
Poor functional outcome	Intercept = 2.11 (1.95–0.34)	0.001	—

### 3.5 Certainty of evidence for key outcomes (GRADE assessment)

The GRADE assessment (Table 5) indicates that the overall certainty of evidence for the main clinical outcomes is moderate for mortality and poor functional status, but low for hematoma expansion. For 30–day and 1–year mortality, most studies had low risk of bias, with no major concerns about indirectness or imprecision; however, serious inconsistency (*I*^2^: >50%) and suspected or likely publication bias limited confidence, yielding a moderate certainty rating. Poor functional outcome (mRS: 4–6) showed a similar pattern: low risk of bias and adequate precision, but high heterogeneity and suspected publication bias, resulting in moderate certainty. In contrast, hematoma expansion was supported by fewer studies with moderate risk of bias, serious heterogeneity, and serious imprecision, leading to low–certainty evidence.

**Table 5:** Quality of evidence (GRADE assessment).

Outcome	No. studies	Risk of bias	Inconsistency	Indirectness	Imprecision	Publication bias	GRADE certainty
30-day mortality	22	Low	Serious^*^	None	None	Suspected	Moderate
1-year mortality	14	Low	Serious	None	None	Likely	Moderate
Hematoma expansion	8	Moderate	Serious	None	Serious	None	Low
Poor functional outcome (mRS 4–6)	16	Low	Serious	None	None	Suspected	Moderate

^*^Serious inconsistency due to I^2^ > 50%.

### 3.6 Study quality assessment

Overall, the methodological quality of the included studies was considered acceptable, although several potential sources of bias were identified. Most reports provided clear case definitions and explicit outcome criteria, which contributed to a relatively low risk of detection bias. However, heterogeneity in patient selection strategies and in the extent of statistical adjustment for confounding factors led to moderate concern regarding selection and comparability bias in a substantial subset of studies. In addition, a minority of studies did not fully describe the duration or completeness of follow–up, introducing some uncertainty in the interpretation of long–term outcomes. The risk–of–bias summary (Supplementary Table S1 and Supplementary Figure S1) indicate that the majority of studies fall within the low–to–moderate risk categories, suggesting that the aggregated findings are based on evidence of generally acceptable quality.

## 4 Discussion

Baseline differences in Table 1 indicate that CKD marks a cluster of adverse host factors rather than an isolated comorbidity in ICH. The older age and high prevalence of hypertension and diabetes mirror the systemic vasculopathy and small–vessel arteriopathy long recognized in CKD populations (10, 28, 29). Deep intracerebral hemorrhages are commonly linked to chronic hypertensive injury affecting small penetrating cerebral arteries, a pathological process characterized by lipohyalinosis and microaneurysm formation within the basal ganglia and thalamic vascular territories. The greater anticoagulant exposure in CKD likely reflects a higher burden of atrial fibrillation and structural heart disease, but may also amplify bleeding risk because several anticoagulants have reduced clearance in impaired renal function, predisposing to larger hematomas and more severe neurological deficits at presentation (29, 30).

These baseline imbalances have important implications for outcome analyses. First, they support viewing CKD as a surrogate for diffuse vascular and hemostatic vulnerability rather than simply reduced eGFR. Second, they highlight the need for rigorous adjustment for age, comorbidities, anticoagulant use, hematoma volume, and ICH location when estimating the independent prognostic effect of CKD. Without such adjustment, any correlation between CKD and poor outcome may be conflated with baseline severity. Finally, the sizeable dialysis–dependent subgroup suggests that future work should separate non–dialysis CKD from end–stage kidney disease, as mechanisms and modifiable risks may differ.

When interpreting the results of this meta-analysis, several methodological limitations of the underlying studies should be taken into account. Differences in study design, inclusion criteria, and outcome assessment protocols likely contributed to the observed heterogeneity in effect estimates (15). In some cohorts, adjustment for key prognostic variables, such as baseline neurological status, hematoma volume, or comorbid cardiovascular and renal conditions, was incomplete or absent, which may have influenced the magnitude and precision of reported associations (11). Despite these constraints, most studies used broadly comparable definitions of CKD and ICH outcomes and adhered to acceptable reporting standards, supporting the overall reliability of the synthesized evidence. Future research would benefit from more standardized protocols, harmonized reporting of prognostic variables, and larger multicenter datasets to refine estimates and reduce residual bias in this field.

The outcome patterns in Table 2 show a clear and clinically important gradient of risk across the kidney disease spectrum. Even mild–to–moderate CKD (eGFR < 60 ml/min/1.73 m^2^) was associated with nearly double the short–term mortality after ICH and almost a three–fold increase in 1–year death, while severe CKD and ESRD showed extremely high fatality rates, exceeding 80% at 1 year. This stage–response relationship supports CKD as a strong, biologically plausible prognostic factor rather than a passive marker of age or comorbidity, consistent with prior work linking CKD to higher stroke mortality and disability (10, 29, 31). Several mechanisms may underlie these findings, including uremic platelet dysfunction, endothelial injury, chronic inflammation, and blood pressure lability, which together can promote larger hematomas, hematoma expansion, and treatment complications (29, 31). The excess of poor functional outcomes (mRS: 4–6) in CKD, even among survivors, suggests that renal dysfunction not only increases mortality but also limits recovery potential, possibly through frailty, sarcopenia, and higher burden of cardiovascular and cerebrovascular disease.

The observed moderate-to-high heterogeneity (*I*^2^ >50% for several outcomes) underscores the underlying clinical and methodological diversity of the included studies and limits the precision of the pooled risk estimates. Variability in CKD staging, dialysis practices, comorbidity burden, and ICH management protocols, as well as differences in follow–up duration and outcome definitions, may all contribute to the inconsistent effect sizes seen across cohorts (14). Rather than contradicting the signal of increased risk, this pattern likely reflects the heterogeneous ways in which CKD influences vascular fragility, hematoma dynamics, and systemic complications in diverse patient populations, and highlights the need for standardized, prospective, multicenter studies to refine these estimates.

**Figure 5:**
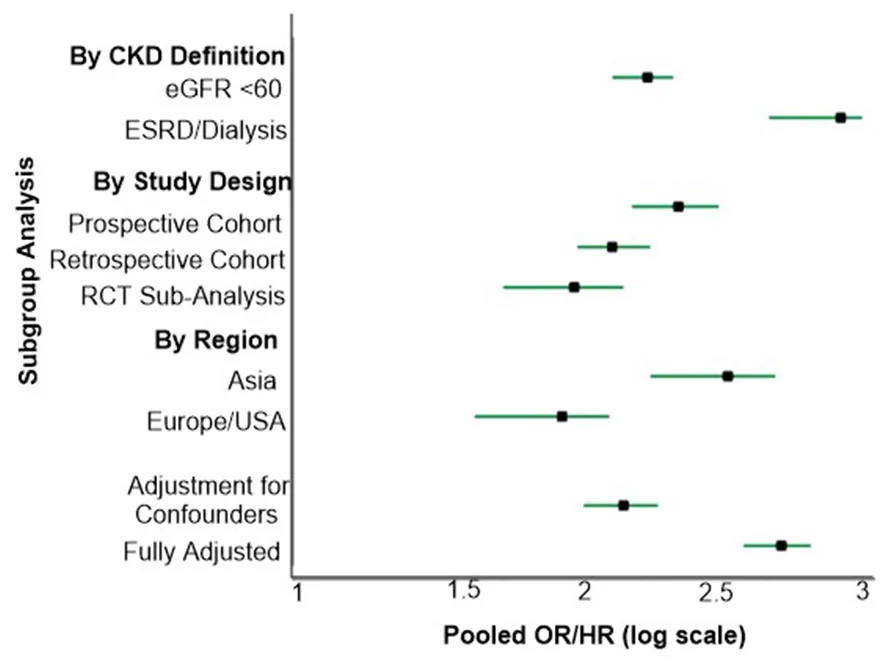
Forest plot of pooled subgroup effects for CKD and outcomes after intracerebral hemorrhage.

The subgroup findings in Table 3 help explain, but do not eliminate, the heterogeneity observed in the primary analyses. The broadly consistent effect across CKD definitions and study designs supports a genuine, independent association between renal dysfunction and worse ICH prognosis, in line with prior work linking reduced eGFR to higher stroke mortality and disability (1, 2). The larger point estimate in ESRD/dialysis, albeit without statistically significant interaction, is clinically coherent with the cumulative burden of uremia, dialysis–related hemodynamic stress, and coagulopathy in advanced kidney failure (29). Regional differences are notable: Asian cohorts showed stronger CKD–outcome associations than European/US studies, Figure 5. This may reflect higher baseline rates of uncontrolled hypertension, differing CKD etiologies, genetic susceptibility to small–vessel disease, and variations in acute ICH management and access to intensive care (28, 31). These factors could magnify the impact of impaired renal function on hemorrhage severity and recovery.

The contrast between fully adjusted and unadjusted/partially adjusted models indicates that age, comorbidities, and ICH characteristics confound part of the association; however, the persistence of a significant effect after rigorous adjustment suggests that CKD exerts additional biological influence. Lower heterogeneity in RCT sub–analyses further implies that standardized protocols and more complete covariate data can reduce variability, underscoring the need for well-phenotyped, prospective CKD–ICH cohorts (32).

**Figure 6:**
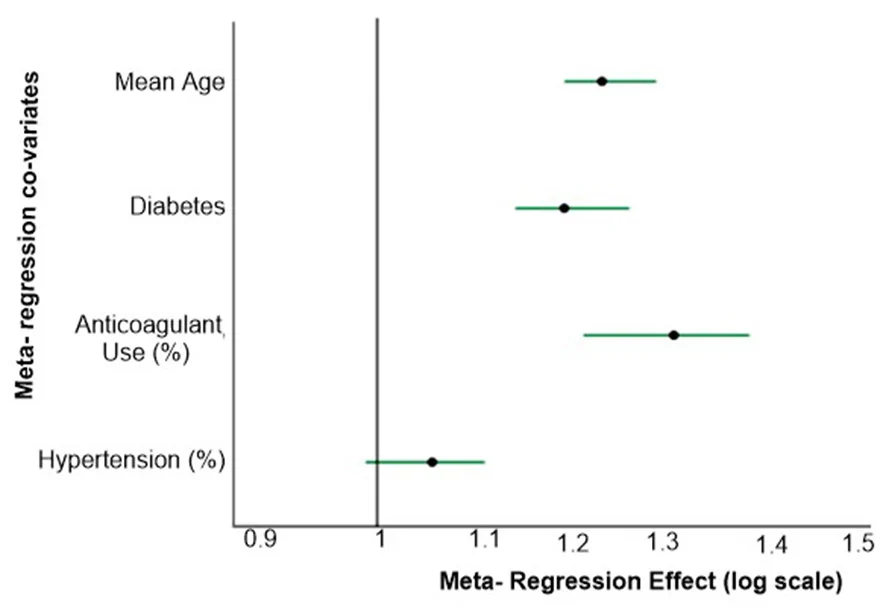
Forest plot of meta-regression covariates associated with outcomes in CKD after intracerebral hemorrhage.

The meta–regression findings in Table 4 suggest that the prognostic impact of CKD after ICH is amplified in older, more comorbid populations and in settings with greater anticoagulant exposure. The positive age coefficient aligns with evidence that renal dysfunction, cerebrovascular fragility, and reduced physiological reserve synergistically worsen stroke outcomes in older adults (28, 29). Likewise, higher study–level diabetes prevalence likely reflects more advanced systemic micro– and macro–vascular disease, providing a plausible substrate for both larger hematomas and impaired recovery. The particularly strong influence of anticoagulant use is consistent with clinical data showing that impaired renal clearance of anticoagulants increases bleeding risk and complicates reversal strategies (29, 33). Figure 6 helps to contextualize the heterogeneity observed across studies by showing which study–level covariates systematically modify the CKD–ICH association. The positive coefficients for mean age, diabetes prevalence, and anticoagulant use suggest that CKD has the greatest prognostic impact in older, more metabolically burdened populations and in settings with frequent anticoagulant exposure.

However, the relatively modest adjusted *R*^2^ values (8%−15%) indicate that these covariates explain only part of the between–study heterogeneity; differences in CKD staging, dialysis practices, imaging protocols, and intensity of acute care probably contribute as well. The significant Egger's test results for 30–day mortality and poor functional outcome raise concern for small–study effects or selective non–publication of neutral findings (34). While such bias may inflate the absolute magnitude of the pooled estimates, the consistency of direction across subgroups and sensitivity analyses makes it unlikely that the CKD–ICH association is solely artefactual. These results argue for cautious interpretation of effect size, but reinforce the need to explicitly account for age, diabetes, and anticoagulation when modeling CKD–related risk.

The GRADE evaluation in Table 5 suggests that the correlation between CKD and “hard” clinical outcomes after ICH short–term mortality, longer–term mortality, and poor functional status is supported by a generally reliable but not definitive body of evidence. Low risk of bias across most mortality and disability studies, combined with direct populations and outcomes, supports the validity of the observed associations (28, 29, 31). However, serious inconsistency (*I*^2^ > 50%) indicates that effect sizes vary meaningfully across settings, likely reflecting differences in CKD case–mix, dialysis practices, and ICH management. Suspected or likely publication bias, particularly for 1–year mortality, further suggests that the true effect may be somewhat smaller than the pooled estimates (35).

The findings of this meta-analysis have several important implications for the management of patients with intracerebral hemorrhage. The consistent association between chronic kidney disease and unfavorable outcomes indicates that renal dysfunction should be considered a key systemic factor when evaluating ICH prognosis, rather than a peripheral comorbidity. Patients with impaired renal function frequently exhibit vascular fragility, endothelial dysfunction, and altered coagulation pathways, which may contribute to hematoma expansion and reduced neurological recovery. Recognizing CKD as a prognostic modifier may assist clinicians in early risk stratification and support more intensive monitoring and closer hemodynamic and anticoagulation management during the acute phase of hemorrhage (11, 36). More broadly, these results highlight the importance of integrating systemic comorbidities into clinical decision-making frameworks that have traditionally emphasized neurological and radiological indicators alone. Improved recognition and documentation of renal dysfunction in this context may facilitate the development of more comprehensive prognostic models and encourage multidisciplinary management strategies involving both neurological and nephrological expertise in patients presenting with ICH and CKD (18, 19).

For hematoma expansion, the downgrade to low certainty reflects both methodological and statistical limitations: fewer contributing studies, moderate risk of bias, and wide confidence intervals prevent firm conclusions. Clinically, this means that while CKD can reasonably be incorporated into prognostic discussions and risk models for mortality and functional dependence, any claims about its specific effect on hematoma dynamics should be considered provisional. Future high–quality, prospective studies with standardized CKD staging, imaging protocols, and comprehensive reporting will be essential to upgrade the certainty of evidence and refine risk stratification.

## 5 Conclusion

Chronic kidney disease emerges from this meta-analysis as a strong, independent predictor of poor outcome after intracerebral hemorrhage. Across approximately 5,000 patients, impaired renal function was consistently linked to higher short–term and 1–year mortality, greater odds of severe functional dependence, and an increased risk of hematoma expansion, with the most pronounced risks seen in individuals with eGFR < 30 ml/min/1.73 m^2^ or receiving dialysis. These associations persisted after adjustment for age, comorbidities, anticoagulant use, and key hemorrhage characteristics, indicating that CKD contributes prognostic information beyond conventional neurological and radiological markers.

Although moderate–to–high heterogeneity and signals of publication bias limit the precision of the pooled estimates, the direction and size of effects were robust across multiple subgroup and sensitivity analyses, and the GRADE assessment supports moderate certainty for mortality and disability outcomes. Taken together, these findings support incorporating renal function into routine ICH risk stratification, prognostic discussions, and decisions about monitoring intensity and resource allocation. CKD should be explicitly considered in clinical pathways and in the design of future prognostic models for ICH. Further prospective, CKD–focused studies and targeted interventional trials are needed to refine risk estimates, clarify mechanisms linking renal dysfunction to hemorrhage progression, and identify modifiable treatment strategies in this high–risk population.

## Data Availability

The original contributions presented in the study are included in the article/Supplementary material, further inquiries can be directed to the corresponding author.
