# Correction: Risk stratification and prognostic outcomes in intracerebral hemorrhage among patients with chronic kidney disease: a population-oriented meta-analysis

**DOI:** 10.3389/fmed.2026.1974101

**Published:** 2026-09-07

**Authors:** Li Yang, Youbao Li, Zhanmei Zhou, Wenhao Zhang, Xiaodong Yang, Wenjiao Wu, Hui Zhou

**Affiliations:** 1 Division of Nephrology, Nanfang Hospital, Southern Medical University, Guangzhou, Guangdong, China; 2 Department of Neurosurgery, The First Affiliated Hospital of Guangdong Pharmaceutical University, Guangzhou, Guangdong, China

**Keywords:** chronic kidney disease, end-stage renal disease, intracerebral hemorrhage, meta-analysis, risk stratification

The funder The President Foundation of Nanfang Hospital, Southern Medical University (No. 2022A013) was erroneously omitted.

The original version of this article has been updated.

