# Supplementary material for: Risk stratification and prognostic outcomes in intracerebral hemorrhage among patients with chronic kidney disease: a population-oriented meta-analysis
Source: Front Med (Lausanne). 2026 Apr 16;13:1759907. doi: 10.3389/fmed.2026.1759907 (PMC13130474; doi:10.3389/fmed.2026.1759907)
Supplement: Supplementary file 1 [file Data_Sheet_1.docx]

**Table S1.** Key characteristics of studies included in the meta-analysis on CKD and intracerebral hemorrhage

| No. | Author (Year) | Design | Country | Sample Size | CKD Definition | Outcomes | NOS / Quality Notes | Ref |
| --- | --- | --- | --- | --- | --- | --- | --- | --- |
| 1 | O'Donnell et al. (2010) | Prospective cohort (INTERSTROKE Study) | International (22 countries) | 3,000 ICH cases | CKD defined by history of kidney disease or elevated creatinine (exact thresholds in methods). | ICH risk factors including hypertension, smoking, alcohol, and medical comorbidities; CKD as one exposure variable. | Large multi-country cohort; standardized protocols; NOS likely ≥8. | (40) |
| 2 | Tung IM et al. (2021) | Retrospective cohort | Belgium | 842 | eGFR <60 | Stroke outcomes, mortality | 7 | (41) |
| 3 | Beuscher VD et al. (2020) | Retrospective cohort | Germany | 1,135 | CKD stages 3–5 | ICH outcomes, hematoma expansion | 8 | (42) |
| 4 | Molshatzki N et al. (2011) | Prospective cohort | Spain | 128 | eGFR <60 | Hematoma volume, poor outcome | 6 | (43) |
| 5 | Li Z et al. (2022) | Prospective cohort | China | 642 | eGFR <60 | Mortality, functional recovery | 7 | (44) |
| 6 | Zheng D et al. (2016) | RCT sub-analysis | Multinational | 1,234 | eGFR <60 | BP lowering effect on ICH outcomes | 8 | (23) |
| 7 | Ovbiagele B et al. (2014) | Retrospective cohort | USA | 4,256 | CKD diagnosis via ICD | Mortality, care patterns | 7 | (22) |
| 8 | Fukuda-Doi M et al. (2021) | RCT sub-analysis (ATACH-2) | Japan | 840 | eGFR <60 | BP lowering, outcome | 8 | (45) |
| 9 | Wang I-K et al. (2023) | Prospective cohort | Taiwan | 1,125 | eGFR <60 | 1-month, 1-year mortality | 7 | (46) |
| 10 | Lui AK et al. (2023) | Retrospective cohort | Australia | 312 ESRD patients | ESRD on dialysis | Mortality, functional outcomes | 6 | (47) |
| 11 | Kim KR & Kim YZ (2013) | Retrospective cohort | South Korea | 276 | ESRD | 30-day mortality, 6-month mRS | 6 | (48) |
| 12 | Xu M et al. (2016) | Retrospective cohort | China | 198 | ESRD | Hematoma volume, IVH | 7 |  |
| 13 | Shimoyama T et al. (2013) | Prospective cohort | Japan | 164 | Hemodialysis | Early mortality | 7 | (49) |
| 14 | Khatri R et al. (2019) | Retrospective cohort | USA | 1,012 | Pre-existing renal failure | In-hospital mortality | 7 | (50) |
| 15 | Sakamoto N et al. (2014) | Prospective cohort | Japan | 142 | Hemodialysis | ICH outcome, functional recovery | 6 | (51) |
| 16 | Nash PS et al. (2025) | Prospective cohort | UK | 1,050 | CKD stages 1–5 | Anticoagulation timing, ICH outcomes | 8 | (52) |
| 17 | Pieniazek A et al. (2021) | Prospective cohort | Poland | 152 | CKD stages 3–5 | Oxidative stress, uremic toxins | 7 | (53) |
| 18 | Mihaela-Roxana G et al. (2025) | Prospective cohort | Romania | 98 | CKD stages 3–5 | Platelet mitochondrial dysfunction | 7 | (54) |
| 19 | Gao W et al. (2025) | Prospective cohort | China | 230 | CKD stages 3–5 | Inflammatory indicators | 7 | (55) |
| 20 | Gimena Muñoz R et al. (2025) | Prospective cohort | Spain | 160 | Advanced CKD | Frailty, microvesicles | 7 | (56) |
| 21 | Huang M-J et al. (2017) | Prospective cohort | China | 300 | CKD stages 3–5 | Coagulation system | 7 | (57) |
| 22 | Saeed et al. (2015) | Retrospective national cohort using NIS database | United States | 614,454 ICH patients (41,694 with ARF after CKD exclusions) | CKD patients were excluded using ICD-9 codes (585.3–585.6, 585.9, transplant codes). Study focused on ARF only, not CKD. | In-hospital mortality, moderate-to-severe disability, need for dialysis, LOS, hospital charges. | High-quality national database; adjusted for major confounders; NOS likely ≥7. | (58) |
| 23 | Lin et al. (2025) | Retrospective Cohort Study | China | 329 | eGFR < 60 mL/min/1.73 m² (KDIGO 2024 criteria); eGFR calculated using CKD-EPI 2009 formula | Primary: All-cause in-hospital mortality. Secondary: Symptomatic intracerebral hemorrhage (sICH), Hemorrhagic transformation (HT). | - | (59) |
| 24 | Zhang et al. (2021) | Retrospective cohort | China | 1449 | Preexisting chronic kidney disease (based on medical history) | Long-term mortality, AKI risk factors | Focus on long-term mortality in ICH patients with and without preexisting CKD. | (60) |
| 25 | Jiang et al. (2019) | Retrospective observational | China | 381 | Baseline eGFR <15 mL/min/1.73 m² or history of ESKD/dialysis | AKI incidence, 28-day mortality, predictive value of serum cystatin C | Evaluated biomarkers for early AKI prediction in stroke patients. | (61) |
| 26 | Watanabe et al. (2024) | Retrospective cohort | Japan | 100 | End-stage kidney disease (ESKD) on hemodialysis | Functional prognosis (mRS, FIM), mortality, predictors of poor outcome | Focus on functional outcomes and dialysis withdrawal in ICH patients on HD. | (62) |
| 27 | Guo et al. (2026) | Multicenter retrospective cohort | China | 8016 | Excluded patients with pre-existing kidney failure or CKD | AKI incidence, 30-day mortality, predictive value of NAR | Introduced Neutrophil-to-Albumin Ratio (NAR) as a novel AKI predictor. | (63) |
| 28 | Zou et al. (2020) | Retrospective cohort | China | 543 | Excluded patients with pre-existing CKD | Stage 3 AKI risk factors, short-term renal recovery | Identified hypernatremia and hyperuricemia as predictors of severe AKI. | (64) |
| 29 | Huang et al. (2025) | Retrospective cohort | China (Data: USA, MIMIC-IV) | 1,233 | Not explicitly defined; serum creatinine used to calculate sACR | In-hospital mortality (IHM), Long-term mortality (LTM), Severe disturbance of consciousness (SDOC) | First study to link serum albumin-to-creatinine ratio (sACR) with IHM, LTM, and SDOC in ICH | (65) |
| 30 | Yang et al. (2025) | Retrospective cohort | China (Data: USA, MIMIC-III) | 2,421 (398 received mannitol) | Acute kidney injury (AKI) defined by KDIGO criteria | AKI, In-hospital mortality, ICU length of stay, RRT requirement | Mannitol use associated with higher AKI and mortality, especially in severe ICH (GCS ≤ 8) | (66) |

**Supplementary Table S2. Risk of Bias Assessment of Included Studies (Newcastle–Ottawa Scale)**

| **Study** | **Selection (4)** | **Comparability (2)** | **Outcome (3)** | **Total Score** | **Risk of Bias** |
| --- | --- | --- | --- | --- | --- |
| O'Donnell et al. (2010) | 4 | 2 | 3 | 9 | Low |
| Tung IM et al. (2021) | 4 | 2 | 3 | 9 | Low |
| Beuscher VD et al. (2020) | 3 | 2 | 3 | 8 | Low |
| Molshatzki N et al. (2011) | 3 | 2 | 3 | 8 | Low |
| Li Z et al. (2022) | 3 | 2 | 2 | 7 | Moderate |
| Zheng D et al. (2016) | 4 | 1 | 3 | 8 | Low |
| Ovbiagele B et al. (2014) | 3 | 2 | 2 | 7 | Moderate |
| Fukuda-Doi M et al. (2021) | 3 | 2 | 3 | 8 | Low |
| Wang I-K et al. (2023) | 3 | 1 | 3 | 7 | Moderate |
| Lui AK et al. (2023) | 4 | 2 | 2 | 8 | Low |
| Kim KR & Kim YZ (2013) | 3 | 2 | 2 | 7 | Moderate |
| Xu M et al. (2016) | 4 | 2 | 3 | 9 | Low |
| Shimoyama T et al. (2013) | 3 | 2 | 2 | 7 | Moderate |
| Khatri R et al. (2019) | 4 | 2 | 3 | 9 | Low |
| Sakamoto N et al. (2014) | 3 | 2 | 2 | 7 | Moderate |
| Nash PS et al. (2025) | 3 | 1 | 3 | 7 | Moderate |
| Pieniazek A et al. (2021) | 4 | 2 | 3 | 9 | Low |
| Mihaela-Roxana G et al. (2025) | 3 | 2 | 3 | 8 | Low |
| Gao W et al. (2025) | 3 | 2 | 2 | 7 | Moderate |
| Gimena Muñoz R et al. (2025) | 4 | 2 | 3 | 9 | Low |
| Huang M-J et al. (2017) | 3 | 2 | 2 | 7 | Moderate |
| Saeed et al. (2015) | 4 | 2 | 3 | 9 | Low |
| Lin et al. (2025) | 3 | 2 | 2 | 7 | Moderate |
| Zhang et al. (2021) | 3 | 1 | 3 | 7 | Moderate |
| Jiang et al. (2019) | 4 | 2 | 3 | 9 | Low |
| Watanabe et al. (2024) | 3 | 2 | 2 | 7 | Moderate |
| Guo et al. (2026) | 3 | 2 | 3 | 8 | Low |
| Zou et al. (2020) | 4 | 2 | 3 | 9 | Low |
| Huang et al. (2025) | 3 | 2 | 2 | 7 | Moderate |
| Yang et al. (2025) | 4 | 2 | 3 | 9 | Low |

Supplementary Figure S1. Risk of bias summary of the included observational studies assessed using the Newcastle–Ottawa Scale. Most studies demonstrated low risk of bias in the domains of cohort selection and outcome assessment, while moderate risk was primarily related to incomplete adjustment for potential confounders.

**References:**

22. Ovbiagele B, Schwamm LH, Smith EE, Grau-Sepulveda M V., Saver JL, Bhatt DL, et al. Hospitalized Hemorrhagic Stroke Patients with Renal Insufficiency: Clinical Characteristics, Care Patterns, and Outcomes. Journal of Stroke and Cerebrovascular Diseases. 2014 Oct;23(9):2265–73. doi:10.1016/j.jstrokecerebrovasdis.2014.04.016

23. Zheng D, Sato S, Arima H, Heeley E, Delcourt C, Cao Y, et al. Estimated GFR and the Effect of Intensive Blood Pressure Lowering After Acute Intracerebral Hemorrhage. American Journal of Kidney Diseases. 2016 Jul;68(1):94–102. doi:10.1053/j.ajkd.2016.01.020

40. O’Donnell MJ, Xavier D, Liu L, Zhang H, Chin SL, Rao-Melacini P, et al. Risk factors for ischaemic and intracerebral haemorrhagic stroke in 22 countries (the INTERSTROKE study): a case-control study. The Lancet. 2010 Jul;376(9735):112–23. doi:10.1016/S0140-6736(10)60834-3

41. Tung IM, Barlas RS, Vart P, Bettencourt-Silva JH, Clark AB, Sawanyawisuth K, et al. Association of chronic kidney disease with outcomes in acute stroke. Acta Neurol Belg. 2021 Oct 13;121(5):1241–6. doi:10.1007/s13760-020-01416-0

42. Beuscher VD, Sprügel MI, Gerner ST, Sembill JA, Madzar D, Reindl C, et al. Chronic Kidney Disease and Clinical Outcomes in Patients with Intracerebral Hemorrhage. Journal of Stroke and Cerebrovascular Diseases. 2020 Aug;29(8):104802. doi:10.1016/j.jstrokecerebrovasdis.2020.104802

43. Molshatzki N, Orion D, Tsabari R, Schwammenthal Y, Merzeliak O, Toashi M, et al. Chronic Kidney Disease in Patients with Acute Intracerebral Hemorrhage: Association with Large Hematoma Volume and Poor Outcome. Cerebrovascular Diseases. 2011;31(3):271–7. doi:10.1159/000322155

44. Li Z, Li Z, Zhou Q, Gu H, Wang Y, Zhao X, et al. Effects of estimated glomerular filtration rate on clinical outcomes in patients with intracerebral hemorrhage. BMC Neurol. 2022 Dec 10;22(1):19. doi:10.1186/s12883-022-02551-2

45. Fukuda-Doi M, Yamamoto H, Koga M, Doi Y, Qureshi AI, Yoshimura S, et al. Impact of Renal Impairment on Intensive Blood-Pressure–Lowering Therapy and Outcomes in Intracerebral Hemorrhage. Neurology. 2021 Aug 31;97(9). doi:10.1212/WNL.0000000000012442

46. Wang IK, Yen TH, Tsai CH, Sun Y, Chang WL, Chen PL, et al. Renal function is associated with one-month and one-year mortality in patients with intracerebral hemorrhage. PLoS One. 2023 Jan 26;18(1):e0269096. doi:10.1371/journal.pone.0269096

47. Lui AK, Lin F, Uddin A, Nolan B, Clare K, Nguyen T, et al. A double-hit: End-stage renal disease patients suffer worse outcomes in intracerebral hemorrhage. Brain Circ. 2023 Jul;9(3):172–7. doi:10.4103/bc.bc_24_23

48. Kim KR, Kim YZ. Clinical Comparison of 30-Day Mortalities and 6-Month Functional Recoveries after Spontaneous Intracerebral Hemorrhage in Patients with or without End-Stage Renal Disease. J Korean Neurosurg Soc. 2013;54(3):164. doi:10.3340/jkns.2013.54.3.164

49. Shimoyama T, Kimura K, Shibazaki K, Yamashita S, Iguchi Y. Maintenance Hemodialysis Independently Increases the Risk of Early Death after Acute Intracerebral Hemorrhage. Cerebrovascular Diseases. 2013;36(1):47–54. doi:10.1159/000351504

50. Khatri R, Afzal MR, Qureshi MA, Maud A, Huanyu D, Jose Rodriguez G. Pre-Existing Renal Failure Increases In-Hospital Mortality in Patients with Intracerebral Hemorrhage. Journal of Stroke and Cerebrovascular Diseases. 2019 Feb;28(2):237–42. doi:10.1016/j.jstrokecerebrovasdis.2018.07.036

51. Sakamoto N, Ishikawa E, Aoki K, Uemae Y, Komatsu Y, Matsumura A. Clinical Outcomes of Intracerebral Hemorrhage in Hemodialysis Patients. World Neurosurg. 2014 Mar;81(3–4):538–42. doi:10.1016/j.wneu.2013.10.033

52. Nash PS, Dehbi HM, Ahmed N, Arram L, Best JG, Balogun M, et al. Anticoagulation Timing in Acute Stroke With Atrial Fibrillation According to Chronic Kidney Disease: The OPTIMAS Trial. Stroke. 2025 Aug;56(8):1970–9. doi:10.1161/STROKEAHA.125.051457

53. Pieniazek A, Bernasinska-Slomczewska J, Gwozdzinski L. Uremic Toxins and Their Relation with Oxidative Stress Induced in Patients with CKD. Int J Mol Sci. 2021 Jun 8;22(12):6196. doi:10.3390/ijms22126196

54. Mihaela-Roxana G, Theia SL, Oana-Maria A, Anca-Mihaela B, Vlad-Florian A, Lavinia B, et al. Impairment of platelet mitochondrial respiration in patients with chronic kidney disease with and without diabetes. Mol Cell Biochem. 2025 Aug 12;480(8):4745–55. doi:10.1007/s11010-025-05280-5

55. Gao W, Wang X, Zou Y, Wang S, Dou J, Qian S. Progress in the application of novel inflammatory indicators in chronic kidney disease. Front Med (Lausanne). 2025 Jan 30;12. doi:10.3389/fmed.2025.1500166

56. Gimena Muñoz R, Valera Arévalo G, Rodríguez San Pedro M del M, Pérez Fernández M, Arévalo Serrano J, S. Waikar S, et al. Assessment of frailty in patients with advanced chronic kidney disease, and the role of microvesicles: A single-center study. PLoS One. 2025 Sep 30;20(9):e0332653. doi:10.1371/journal.pone.0332653

57. Huang MJ, Wei R bao, Wang Y, Su T yu, Di P, Li Q ping, et al. Blood coagulation system in patients with chronic kidney disease: a prospective observational study. BMJ Open. 2017 May 1;7(5):e014294. doi:10.1136/bmjopen-2016-014294

58. Saeed F, Adil MM, Piracha BH, Qureshi AI. Acute Renal Failure Worsens In-hospital Outcomes in Patients with Intracerebral Hemorrhage. Journal of Stroke and Cerebrovascular Diseases. 2015 Apr;24(4):789–94. doi:10.1016/j.jstrokecerebrovasdis.2014.11.012

59. Lin Y, She J, Cai L, Yu L, Jin S, Chen X, et al. Dynamic decline in estimated glomerular filtration rate associated with in-hospital mortality risk in acute ischemic stroke patients after endovascular therapy: evidence from a Chinese stroke center. Front Aging Neurosci. 2025 Nov 6;17. doi:10.3389/fnagi.2025.1598371

60. Zhang C, Xia J, Ge H, Zhong J, Chen W, Lan C, et al. Long-Term Mortality Related to Acute Kidney Injury Following Intracerebral Hemorrhage: A 10-Year (2010–2019) Retrospective Study. Journal of Stroke and Cerebrovascular Diseases. 2021 May;30(5):105688. doi:10.1016/j.jstrokecerebrovasdis.2021.105688

61. Jiang F, Su L, Xiang H, Zhang X, Xu D, Zhang Z, et al. Incidence, Risk factors, and Biomarkers Predicting Ischemic or Hemorrhagic Stroke Associated Acute Kidney Injury and Outcome: A Retrospective Study in a General Intensive Care Unit. Blood Purif. 2019;47(4):317–26. doi:10.1159/000499029

62. Watanabe Y, Suzuki K, Inoue T, Kurita H, Okada H. Functional prognosis following spontaneous intracerebral hemorrhage in patients on hemodialysis: a retrospective study of 100 consecutive cases. Ren Replace Ther. 2024 Mar 4;10(1):12. doi:10.1186/s41100-024-00528-0

63. Guo Q, Zhou K, Li Y, Wan J, Yang F, Fang F, et al. Predictive value of neutrophil-to-albumin ratio for the incidence of acute kidney injury in intracerebral hemorrhage patients. Neurosurg Rev. 2025 Nov 21;49(1):20. doi:10.1007/s10143-025-03952-w

64. Zou Z, Chen S, Li Y, Cai J, Fang Y, Xie J, et al. Risk factors for renal failure and short-term prognosis in patients with spontaneous intracerebral haemorrhage complicated by acute kidney injury. BMC Nephrol. 2020 Dec 29;21(1):311. doi:10.1186/s12882-020-01949-9

65. Huang Y, Jing S, Zhang Y, Li J, Wang Z, Zheng W, et al. Association between the serum albumin-to-creatinine ratio and prognosis and risk of severe disturbance of consciousness in intracerebral hemorrhage patients. Sci Rep. 2025 Nov 25. doi:10.1038/s41598-025-29526-w

66. Yang X, Liu Z, Chen X, Deng S, Wang Z, Jin J. Renal impairment and prognosis in mannitol-treated intracerebral hemorrhage: a retrospective clinical analysis. Journal of Clinical Neuroscience. 2025 Dec;142:111695. doi:10.1016/j.jocn.2025.111695
